# Routine Surveillance of Upper Urinary Tract Imaging for Diagnosing Upper Urinary Tract Urothelial Cancer Recurrence in Patients with Nonmuscle Invasive Bladder Cancer

**DOI:** 10.1155/2024/5894288

**Published:** 2024-05-21

**Authors:** Nobutaka Nishimura, Makito Miyake, Tatsuki Miyamoto, Takuto Shimizu, Tomomi Fujii, Yosuke Morizawa, Shunta Hori, Daisuke Gotoh, Yasushi Nakai, Kazumasa Torimoto, Nobumichi Tanaka, Kiyohide Fujimoto

**Affiliations:** ^1^Department of Urology, Nara Medical University, 840 Shijo-cho, Kashihara, Nara 634-8522, Japan; ^2^Department of Diagnostic Pathology, Nara Medical University, 840 Shijo-cho, Kashihara, Nara 634-8522, Japan; ^3^Department of Prostate Brachytherapy, Nara Medical University, 840 Shijo-cho, Kashihara, Nara 634-8522, Japan

## Abstract

**Background:**

Although routine surveillance imaging to examine upper urinary tract urothelial cancer recurrence during follow-up of nonmuscle invasive bladder cancer is recommended, its necessity remains invalidated. A single-institute long-term follow-up cohort study to evaluate the clinical impact of routine surveillance imaging and identify risk factors for upper urinary tract urothelial cancer recurrence after nonmuscle invasive bladder cancer treatment was conducted.

**Methods and Materials:**

A retrospective chart review of 864 patients with primary nonmuscle invasive bladder cancer who underwent initial transurethral resection of bladder tumor between 1980 and 2020 was conducted. The opportunities to diagnose its recurrence were examined. Moreover, oncological outcomes included upper urinary tract urothelial cancer recurrence-free survival and overall survival.

**Results:**

Of 864 patients, 19 (2.2%) experienced upper urinary tract urothelial cancer recurrence. Among 19 patients, recurrence was detected through routine imaging in 12 (63.2%), cystoscopy in 2 (10.5%), urine cytology in 2 (10.5%), and presence of gross hematuria in 1 (5.3%). All patients had high- or highest-risk NMIBC at diagnosis of primary nonmuscle invasive bladder cancer. On multivariate Fine-Gray proportional regression analyses, a tumor size of ≥30 mm and carcinoma in situ were independently associated with short upper urinary tract urothelial cancer recurrence-free survival (*P*=0.040 and 0.0089, respectively).

**Conclusion:**

Most patients experiencing upper urinary tract urothelial cancer recurrence were diagnosed by routine surveillance imaging, suggesting its clinical importance, especially for patients with nonmuscle invasive bladder cancer accompanied by a tumor size of ≥30 mm and carcinoma in situ.

## 1. Introduction

Compared to bladder recurrence, upper urinary tract urothelial cancer (UTUC) recurrence during surveillance of nonmuscle invasive bladder cancer (NMIBC) is relatively rare, with an incidence of 2.6–5.8% [1–4]. It is well-known that the risk factors for intravesical recurrence or progression to invasive bladder cancer are associated with high-risk NMIBC, carcinoma in situ (CIS), Bacillus Calmette-Guérin (BCG) resistance, and age [5, 6]. Furthermore, the relationship between various molecular subtypes and bladder cancer has been demonstrated [7]. Previous reports also have described multiplicity, high-risk NMIBC, CIS, vesicoureteral reflux, and failed intravesical chemotherapy as prognostic factors for UTUC recurrence after treatment of NMIBC [8–10].

Each guideline mentions the need for follow-up for NMIBC with regards to the observation of the upper urinary tract.  Table 1 summarizes the follow-up strategies for the diagnosis of UTUC using upper urinary tract imaging. Routine surveillance imaging, including computed tomography urography (CTU), is recommended for patients with high-risk NMIBC but not for those with low or intermediate risk [11–15]. Although CTU has excellent diagnostic performance for UTUC, a risk of contrast nephropathy and radiation exposure exists. Sternberg et al. reported that most UTUC recurrences in patients with NMIBC were missed on routine surveillance imaging, which suggests that this method may not be efficient to diagnose UTUC recurrence [16]. Some authors reported that routine investigations detected 38–78% of UTUC recurrences, even in cohorts of patients after cystectomy [17–19]. The necessity of routine surveillance imaging for diagnosing UTUC after treating bladder cancer remains unclear. If routine imaging for UTUC recurrence is not needed, as described by Steinberg et al., expensive and radiation-exposed examinations that may have adverse effects on both the healthcare system and the human body should be avoided. A single-institute, long-term follow-up cohort study to determine the necessity of routine surveillance imaging in a real-world clinical setting and to identify the risk factors for UTUC recurrence after NMIBC treatment was conducted.

## 2. Materials and Methods

### 2.1. Patient Selection and Study Design

The study protocol was approved by the Institutional Review Board for Clinical Studies (Medical Ethics Committee ID: 2891) at the Nara Medical University in Nara, Japan. The opt-out method was applied to obtain consent from the participants via posters and/or websites, and the study was conducted in compliance with the principles of the Declaration of Helsinki. A preprint of this manuscript has previously been published [20].

A retrospective chart review of 913 patients diagnosed with primary NMIBC who underwent transurethral resection of bladder tumor (TURBT) between 1980 and 2020 was conducted. The inclusion criteria were as follows: (1) primary NMIBC at diagnosis at the Nara Medical University, (2) UTUC recurrence after initial TURBT, and (3) complete clinicopathological data.

The following clinicopathological variables on prognosis were evaluated: age, sex, Eastern Cooperative Oncology Group performance status (ECOG-PS), tumor size, multiplicity, clinical T category, World Health Organization (WHO) 1973 and 2004 grades, CIS, prostate-involving CIS, lymphovascular invasion (LVI), variant histology, risk classification, and intravesical recurrence after initial TURBT. The Clinical T category was determined on imaging before TURBT and through the pathological diagnosis of TURBT. After the advent of the WHO 2004 schemes, the pathologists at our hospital used the WHO 2004 grade instead of the WHO 1973 grade. The risk classification of NMIBC was defined in the Clinical Practice Guidelines for Bladder Cancer 2019 [14]. The criteria for highest-risk NMIBC are as follows: (1) T1 and high grade (HG) tumors with concomitant bladder CIS, concomitant prostate-involving CIS, multiple tumors, recurrent tumors, a tumor size of ≥30 mm, variant histology, and/or LVI and (2) BCG-unresponsive disease. BCG-unresponsive disease was also defined in the Clinical Practice Guidelines for Bladder Cancer 2019 [14].

### 2.2. Follow-Up

Patients were followed up after the initial TURBT according to the protocols of the Nara Medical University. Cystoscopy was performed three months after the initial TURBT. Routine cystoscopy, blood examination, and urine cytology for diagnosing intravesical recurrence were performed every three, six, or 12 months for up to ten years according to the clinicopathological factors. To diagnose the UTUC recurrence, routine radiographic imaging examinations were performed every 6 or 12 months after initial TURBT and further intensified by each attending physician according to the patient's symptoms.

### 2.3. Outcomes

All opportunities to diagnose UTUC recurrence were examined. Oncological outcomes included the UTUC recurrence-free survival and overall survival (OS). UTUC recurrence-free survival OS were defined as the duration from initial TURBT.

### 2.4. Statistical Analysis

Quantitative variables are summarized as means (standard deviation (SD)), and categorical variables are presented as proportions. Student's *t*-test, Fisher's exact test, and the chi-square test were used as appropriate for the statistical analyses of patient clinicopathological characteristics. Prognostic factors for UTUC recurrence-free survival were assessed using a competing risk analysis by the Gray test for cumulative incidence. Competing events were defined as deaths without UTUC recurrence. Univariate and multivariate Fine-Gray competing risk regression analyses were used to estimate the hazard ratios for UTUC recurrence-free survival. The OS was assessed using Kaplan-Meier survival curves constructed using GraphPad Prism 7.0 (GraphPad Software, San Diego, CA, USA). Survival curves were compared using the log-rank test. A *p* value <0.05 was considered statistically significant. Statistical analyses were performed using EZR (Saitama Medical Center, Jichi Medical University, Saitama, Japan) [21].

## 3. Results

### 3.1. Characteristics of Patients with UTUC Recurrence

**Figure 1:**
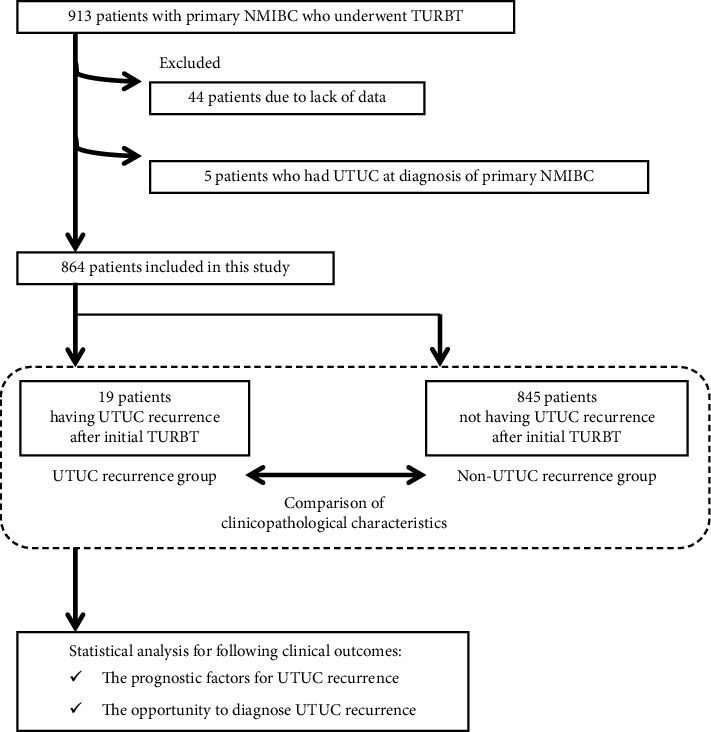
Flowchart of the creation of the patient's cohort dataset. A retrospective chart review of 913 patients diagnosed with primary NMIBC who underwent TURBT between 1980 and 2020 was conducted. Forty-four patients were excluded due to lack of data, and five patients were excluded because they had UTUC at the time of diagnosis with primary NMIBC. Ultimately, 864 patients were included in the analysis.

Figure 1 presents the flowchart of the creation of the patient cohort dataset. Forty-four patients were excluded due to lack of data, and five patients were excluded as they were diagnosed with UTUC at the same time as primary NMIBC. Ultimately, 864 patients were included in the analysis.  Table 2 shows the clinicopathological characteristics of patients and a comparison between the UTUC recurrence and nonUTUC recurrence groups. Of the 864 patients, 19 (2.2%) experienced an UTUC recurrence after initial TURBT for primary NMIBC. The mean (standard deviation) follow-up period from initial TURBT was 58.1 (46.8) months in the UTUC recurrence group and 74.7 (70.6) months in the nonUTUC recurrence group, respectively. A tumor size of ≥30 mm was associated with a risk of UTUC recurrence. In the UTUC recurrence group, zero cases of cTa, four cases of cTis (21.1%), and 15 cases of cT1 (78.9%) were observed. Moreover, there were significantly more patients with the WHO 1973 grade 3, WHO 2004 high grade, and CIS-positive NMIBC in the UTUC recurrence group than in the nonUTUC recurrence group. 16 (84.2%) of the 19 patients in the UTUC recurrence group received intravesical BCG therapy. 8 (42.1%) and 11 (57.9%) of the 19 patients in the UTUC recurrence group had high- or highest-risk NMIBC, respectively.

### 3.2. Clinicopathological Characteristics of 19 Patients Experiencing UTUC Recurrence

**Figure 2:**
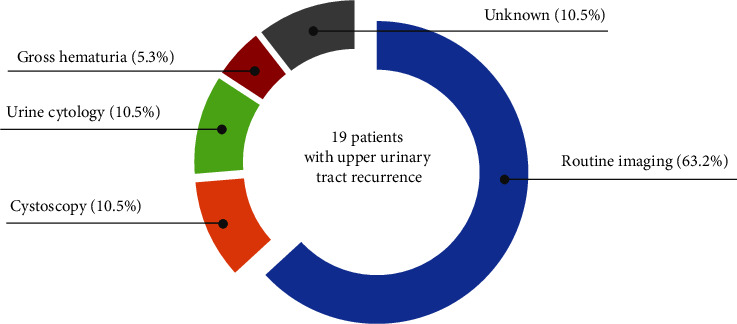
Pie chart of the opportunities to diagnose UTUC recurrence. The opportunities to diagnose UTUC recurrence in UTUC recurrence group are drawn. These are divided into routine imaging, cystoscopy, urine cytology, gross hematuria and unknown.

Among the aforementioned 19 patients, UTUC was diagnosed through routine imaging in 12 (63.2%), cystoscopy in 2 (10.5%), urine cytology in 2 (10.5%), presence of gross hematuria in 1 (5.3%), and unknown methods in 2 (10.5%) patients (Figure 2).  Table 3 summarizes the opportunities for diagnosing UTUC recurrence, clinicopathological characteristics of primary NMIBC, and pathological T categories of UTUC in radical nephroureterectomy specimens or partial ureterectomy specimens. No patient with Ta NMIBC or low-/intermediate-risk NMIBC experienced UTUC recurrence during the follow-up. Of 19 patients in the UTUC recurrence group, 16 (78.9%) had a history of T1 HG NMIBC, 3 (21.1%) had a history of Tis NMIBC, 8 (42.1%) had high-risk NMIBC, and 11 (57.9%) had a highest-risk NMIBC. 16 patients (84.2%) received an intravesical BCG therapy after initial TURBT. Moreover, 16 patients (84.2%) underwent radical operations without neoadjuvant chemotherapy for recurrent lesions of the upper urinary tract. Of these patients, two had pathologically diagnosed pT3 (11.8%), seven had pT2 (41.2%), four had pT1 (23.5%), one had pTis (5.9%), and two had pTa (11.8%). One patient received neoadjuvant chemotherapy and was pathologically diagnosed with ypT0. Three patients were diagnosed with a UTUC recurrence more than ten years after initial TURBT.

### 3.3. Prognostic Factors for UTUC Recurrence

**Figure 3:**
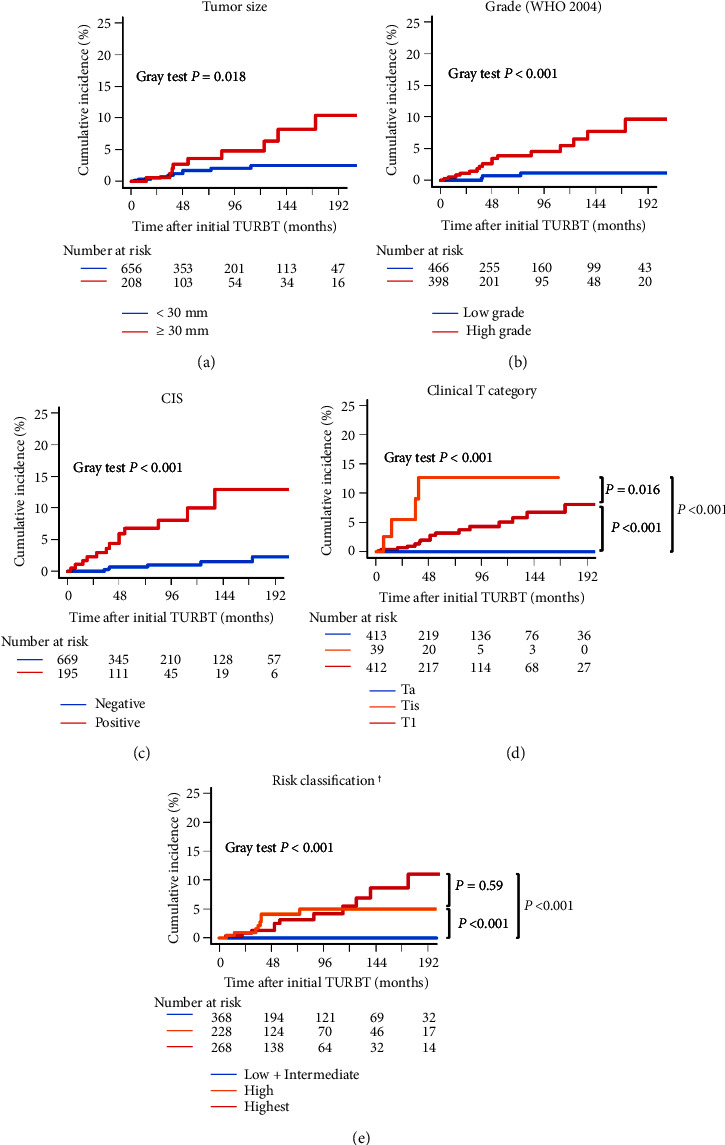
Cumulative incidence of UTUC recurrence-free survival after initial TURBT according to the tumor size, WHO 2004 grade, CIS, clinical T category, and risk classification. Survival curves of the cumulative incidence of UTUC recurrence-free survival after initial TURBT for primary NMIBC were plotted according to the tumor size (a), WHO 2004 grade (b), CIS (c), clinical T category (d), and risk classification (e) using the Gray test. ^†^The risk was classiNed according to the Clinical Practice Guidelines for Bladder Cancer 2019 [14].

**Figure 4:**
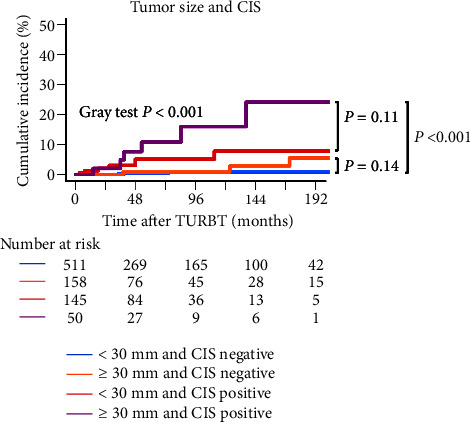
The cumulative incidence of UTUC recurrence-free survival after initial TURBT according to tumor size and CIS. Survival curves of the cumulative incidence of UTUC recurrence-free survival after initial TURBT for primary NMIBC were plotted according to the tumor size and CIS. Patients are categorized into four groups based on whether the tumor size is 30 mm or larger and the presence of CIS.

Figure 3 and Supplementary Figure S1 show the cumulative incidence of UTUC recurrence-free survival after initial TURBT according to the clinicopathological variables. Figure 3 demonstrates that a tumor size of ≥30 mm, high grade, CIS-positive NMIBC, cTis or cT1, and high- or highest-risk NMIBC were associated with a shorter UTUC recurrence-free survival.  Figure 3(d) shows that there was a significant difference in UTUC recurrence for each T category. Furthermore, Figure 3(e) shows no significant difference in UTUC recurrence between high- and highest-risk NMIBC groups. However, high- or highest-risk NMIBC group was significantly associated with a shorter UTUC recurrence-free survival than the low-and intermediate-risk NMIBC group.  Table 4 shows the univariate and multivariate Fine-Gray competing risk regression analyses for UTUC recurrence-free survival.  Figure 4 shows the cumulative incidence of UTUC recurrence-free survival in the four groups categorized according to the tumor size and CIS status. Patients with both a tumor size of ≥30 mm and CIS-positive NMIBC had a significantly shorter UTUC-recurrence-free survival than those lacking these two variables. Multivariate analyses revealed that a tumor size of ≥30 mm (hazard ratio (HR): 2.53; 95% confidence interval (CI): 1.04–6.15; and Fine-Gray *P*=0.040) and CIS-positive NMIBC (HR: 4.78; 95% CI: 1.48–15.42; and Fine-Gray *P*=0.0089) were independently associated with a short UTUC recurrence-free survival.

### 3.4. OS for UTUC Recurrence

**Figure 5:**
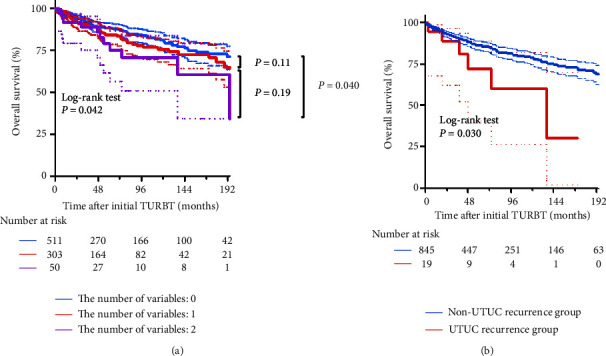
Survival curves for OS after initial TURBT according to the number of prognostic factors and UTUC recurrence. Survival curves for OS after initial TURBT for primary NMIBC are plotted according to the number of prognostic factors (a) and UTUC recurrence (b). Prognostic factors included a tumor size of ≥30 mm and CIS-positive NMIBC. In (a), patients are stratified based on the number of prognostic factors.

Figure 5 compares the survival curves for OS among patients stratified according to the number of risk factors for UTUC recurrence (A) and between those with or without UTUC recurrence (B). Prognostic factors included a tumor size of ≥30 mm and CIS-positive NMIBC. Patients with both prognostic factors had significantly shorter OS (log-rank *P*=0.042) than those without. Moreover, UTUC recurrence was significantly associated with a shorter OS (HR: 3.95; 95% CI: 1.15–13.65; and log-rank *P*=0.030).

## 4. Discussion

It has been reported that the routine surveillance imaging for diagnosing UTUC recurrence after the treatment with NMIBC is not necessary [16, 22]. Sternberg et al. reported that routine imaging identified UTUC recurrences in 4 (25%) out of 16 patients with Ta bladder cancer and 9 (27%) out of 33 patients with T1 bladder cancer. There were no differences in the patient cohort regarding deviation of T category compared to our cohort. In contrast to their findings, our study showed that among 19 patients with UTUC recurrence, the majority (84.2%) were diagnosed by routine surveillance imaging, cystoscopy, or urine cytology and only one (5.3%) was diagnosed by the presence of symptoms (Figure 2 and Table 3). This discrepancy in these results might potentially be attributed to differences of physician's awareness, with a tendency for patients with T1 tumors to be received more cautiously than those with Ta tumors. Moreover, several cases were found to present with invasive urothelial cancer, and 9 cases (47.4%) in the UTUC recurrence group had progressed to muscle invasion disease. If routine surveillance imaging was not performed in these patients, unresectable or metastatic disease may have developed. Moreover, UTUC recurrence increased all-cause mortality (Figure 5(b)) and, therefore, routine surveillance imaging might be necessary to reliably detect upper urinary tract recurrence at an early stage. Most guidelines recommend imaging examination to detect UTUC recurrence for high-risk NMIBC, while they suggest no such procedure for low- or intermediate-risk NMIBC. However, there are few reports to actually evaluate the follow-up strategy for detecting UTUC recurrence. In such a situation, our analysis shows the same conclusion because all patients in the UTUC recurrence group had high- or highest-risk NMIBC, supporting the guidelines' recommendation.

However, it is also considered that it may not be applicable to all patients. The overall incidence of UTUC recurrence in our study was very low at 2.2%, which was almost the same as that of previous reports [1–4], and all patients with UTUC recurrence had high- or highest-risk NMIBC (Table 3). In other words, among patients with high- or highest-risk NMIBC, only a handful does not experience UTUC recurrence. Among these patients, criteria are needed to select those requiring more careful follow-up. Figures 4 and 5 show that a tumor size of ≥30 mm and CIS-positive NMIBC increased the UTUC recurrence and all-cause mortality. Previous reports also described that a history of CIS-positive NMIBC developed aggressive UTUC and increased the cancer-related mortality rate after radical nephroureterectomy for UTUC [23]. Consequently, it is advisable to use tumor size and CIS as these criteria. Even when all patients with high-risk NMIBC undergo imaging examinations of the upper urinary tract, the detection rate for UTUC recurrence remains low. Therefore, we believe it is crucial to pay close attention to those with the highest-risk NMIBC.

**Figure 6:**
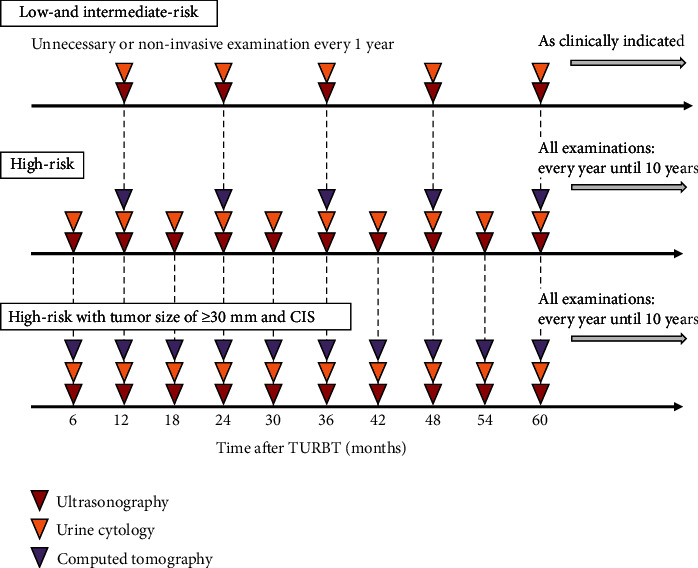
An original follow-up protocol for UTUC recurrence. We have summarized our original devised follow-up protocol for UTUC recurrence. For high-risk NMIBC, the authors recommend that imaging examinations, including CTU, are conducted annually until the fifth year. For high-risk NMIBC with a tumor size of ≥30 mm and CIS, the authors recommend six-month intervals of monitoring up to the fifth year. Routine surveillance is unnecessary for low- and intermediate-risk NMIBC, but noninvasive examinations such as ultrasonography and urine cytology may be performed as needed.

In contrast, there were no patients with low- and intermediate-risk NMIBC who experienced a UTUC recurrence (Table 3). Previous reports have also described the similar results [7–10]. For patients with low- or intermediate-risk NMIBC, follow-up with ultrasonography and urine cytology, which are non-invasive, without radiographic imaging, such as CTU, might be sufficient and cost-saving. Our thoughts on the follow-up protocol for UTUC recurrence are summarized in Figure 6. For high-risk NMIBC, imaging examinations, including CTU, should be conducted annually until the fifth year, as is customary. As previously mentioned, a tumor size of ≥30 mm and CIS were found to be significantly independent risk factors for UTUC recurrence, and thus we believe that the management of the highest-risk NMIBC cases with a tumor size of ≥30 mm and CIS warrants careful consideration. Furthermore, for high-risk NMIBC patients with a tumor size of ≥30 mm and CIS, we recommend six-month intervals of monitoring up to the fifth year rather than twelve-month intervals recommended by many guidelines. On the other hand, the accuracy of CTU and urine cytology are limited [11–13]. DNA methylation analysis can be recently utilized to improve the accuracy to detect UTUC recurrence [24, 25]. In the case of UTUC detection, samples of blood and urine are used, from which DNA is extracted and the methylation patterns of specific gene regions within the DNA are analyzed. This approach may offer superior accuracy to CTU or urine cytology, with less invasiveness for patients than ureteroscopy. Incorporating this innovative analysis in the future could be significantly simplify the detection of UTUC recurrence.

This study has several limitations. First, data on patients were collected retrospectively, and a potential selection bias may have occurred. For example, this cohort included cases from 1980 onwards. To date, the diagnostic abilities of radiographic imaging and endoscopic techniques have advanced, and the follow-up has not been constant between cases. There is a significant difference in image quality and resolution of CT scans with improvements in image reconstruction techniques. Lower image quality of CT scans may result in missing of small lesions. In addition, the decrease of radiation exposure and the cost of examinations have alleviated the burden on patients undergoing tests. This might affect the ability to detect UTUC recurrences because the follow-up protocol intervals have varied between 1980 and 2020. It is challenging to accurately evaluate these data unless these intervals are not standardized. Second, the cohort of UTUC recurrence was too small and there was the significant disparity in patient numbers between the UTUC recurrence and nonUTUC recurrence groups. Among 19 patients in the UTUC recurrence group, it is possible that a normal distribution was not observed in either variable due to the limited sample size. Furthermore, the impact of individual cases becomes magnified in such a small cohort. For instance, if only one out of the 19 patients was detected by the different examination, the recurrence rate could change to 5.3%, which is a significant alteration that could impact the comprehension of these data. Third, due to insufficient data, we were unable to categorize patients into more appropriate groups as follows: those receiving routine surveillance imaging and those not receiving it. As a result, all patients did not undergo this imaging, potentially introducing a significant selection bias. Fourth, intravesical BCG therapy mostly depends on each attending physician and is not uniformly administered for CIS-positive and high-risk NMIBC. Moreover, the number of cases with UTUC recurrence was so small that the HR of intravesical BCG therapy after TURBT could not be calculated, and its impact could not be assessed. Fifth, since multiple surgeons performed TURBT, prognosis might have been influenced by differences in skill. Further prospective cohort studies are required to substantiate the findings of this study. Sixth, there is the uncertain association between vesicoureteral reflex (VUR) and UTUC recurrence. Resecting tumors located around ureteral orifice during TURBT may inadvertently disrupt the vesicoureteral junction, potentially causing the migration of cancer cells to the upper urinary tract. However, due to in sufficient data regarding the resection area during TURBT, we are unable to evaluate this relationship.

## Figures and Tables

**Table 1:** Follow-up strategies for diagnosing UTUC after treatment for NMIBC by upper urinary tract imaging.

Guidelines	Risk classification of each guideline		
	Low	Intermediate	High
AUA/SUO [11]	Unnecessary	Every 1-2 years	Every 1-2 years
CUA [12]	Not mentioned	Not mentioned	Every 1-2 years
EAU [13]	Not mentioned	Not mentioned	Every 1 years
JUA [14]	As clinically indicated	As clinically indicated	Every year for up to 3 years and thereafter every 2 years
NCCN [15]	As clinically indicated	As clinically indicated	At 12 months and, therefore, every 1-2 years

AUA/SUO: American Urological Association/Society of Urologic Oncology; CUA: Canadian Urological Association; EAU: European Association of Urology; JUA: Japanese Urological Association; NCCN: National Comprehensive Cancer Network; NMIBC: nonmuscle invasive bladder cancer; UTUC: upper urinary tract urothelial cancer.

**Table 2:** Clinicopathological characteristics and comparison between the UTUC recurrence group and the non-UTUC recurrence group.

Variables	UTUC recurrence group	Non-UTUC recurrence group	*P* value
	*n* (%)	*n* (%)	
*N*	19 (2.2%)	845 (97.8%)	
Period of follow-up from initial TURBT, mean ± SD	58.1 ± 46.8	74.7 ± 70.6	0.31
Age at initial TURBT, years, mean ± SD	71.6 ± 5.9	76.5 ± 11.9	0.078
Sex			
Male	16 (84.2%)	727 (86.0%)	
Female	3 (15.8%)	118 (14.0%)	
ECOG-PS			1
0-1	19 (100.0%)	828 (98.0%)	
≥2	0 (0.0%)	17 (2.0%)	
Smoking history			1.00
Never	14 (73.7%)	611 (72.3%)	
Former/Current	5 (26.3%)	227 (26.9%)	
Unknown	0 (0.0%)	7 (0.8%)	
Tumor size			0.026
<30 mm	10 (52.6%)	646 (76.4%)	
≥30 mm	9 (47.4%)	199 (23.6%)	
Multiplicity			0.64
Single	12 (63.2%)	474 (56.1%)	
Multiple	7 (36.8%)	371 (43.9%)	
Clinical T category			<0.001
Ta	0 (0.0%)	413 (48.9%)	
Tis	4 (21.1%)	35 (4.1%)	
T1	15 (78.9%)	397 (47.0%)	
Grade (WHO 1973)			<0.001
Grade 1	1 (5.3%)	164 (19.4%)	
Grade 2	2 (10.5%)	406 (48.0%)	
Grade 3	16 (84.2%)	275 (32.5%)	
Grade (WHO 2004)			0.001
Low grade	3 (15.8%)	463 (54.8%)	
High grade	16 (84.2%)	382 (45.2%)	
CIS			<0.001
Negative	6 (31.6%)	663 (78.5%)	
Positive	13 (68.4%)	182 (21.5%)	
Prostate-involving CIS			0.44
Negative	18 (94.7%)	820 (97.0%)	
Positive	1 (5.3%)	25 (3.0%)	
LVI			0.058
Negative	14 (73.7%)	750 (88.8%)	
Positive	5 (26.3%)	95 (11.2%)	
Variant histology			0.46
Negative	18 (94.7%)	819 (96.9%)	
Positive	1 (5.3%)	26 (3.1%)	
Intravesical therapy after TURBT			<0.001
None	3 (15.8%)	473 (56.0%)	
BCG	16 (84.2%)	239 (28.3%)	
IPIC	0 (0.0%)	116 (13.7%)	
BCG + IPIC	0 (0.0%)	17 (2.0%)	
Risk classification^†^			<0.001
Low	0 (0.0%)	186 (22.0%)	
Intermidiate	0 (0.0%)	182 (21.5%)	
High	8 (42.1%)	220 (26.0%)	
Highest	11 (57.9%)	257 (30.4%)	

BCG: Bacillus Calmette-Guérin; CIS: carcinoma in situ; ECOG-PS: Eastern Cooperative Oncology Group performance status; IPIC: immediate postoperative instillation of chemotherapy; LVI: lymphovascular invasion; SD: standard deviation; TURBT: transurethral resection of bladder tumor; UTUC: upper urinary tract urothelial carcinoma; WHO: World Health Organization. ^†^The risk was classified according to the Clinical Practice Guidelines for Bladder Cancer 2019 [14].

**Table 3:** A summary of the opportunities to diagnose UTUC recurrence, clinicopathological characteristics of primary NMIBC, and pathological T categories of UTUC.

Patients	The opportunities of UTUC diagnosis	Clinical T category of primary NMIBC	Grade (WHO 2004)	CIS	Risk classification^†^	Intravesical therapy after initial TURBT	Treatment for UTUC	Pathological T category of UTUC	Periods for UTUC recurrence
1	Routine imaging	T1	HG	Negative	Highest	BCG	Operation	pTa	171
2	Routine imaging	T1	HG	Positive	Highest	BCG	Operation	pT2	136
3	Routine imaging	T1	HG	Negative	Highest	BCG	Operation	pT2	123
4	Urine cytology	T1	HG	Positive	Highest	BCG	Operation	pT2	111
5	Cystoscopy	T1	HG	Positive	Highest	BCG	Operation	pT1	84
6	Gross hematuria	T1	LG	Negative	High	None	Operation	pTa	74
7	Unknown	T1	HG	Positive	Highest	BCG	Operation	pT1	53
8	Urine cytology	T1	HG	Positive	Highest	BCG	BCG into upper urinary tract	Unknown	48
9	Routine imaging	T1	HG	Positive	Highest	BCG	Operation	pT2	48
10	Routine imaging	T1	LG	Negative	High	None	Operation	pT1	39
11	Routine imaging	Tis	HG	Positive	High	BCG	Unknown	Unknown	39
12	Routine imaging	T1	LG	Negative	High	BCG	Operation	pT3	38
13	Routine imaging	Tis	HG	Positive	High	BCG	Operation	pT2	36
14	Routine imaging	T1	HG	Negative	High	BCG	Operation	pT2	34
15	Routine imaging	T1	HG	Positive	Highest	BCG	Operation	pT2	27
16	Unknown	T1	HG	Positive	Highest	None	Operation	pT3	18
17	Routine imaging	Tis	HG	Positive	High	BCG	Operation	pT0 after NAC	14
18	Routine imaging	Tis	HG	Positive	High	BCG	Operation	pTis	7
19	Cystoscopy	T1	HG	Positive	Highest	BCG	Operation	pT1	3

BCG: Bacillus Calmette-Guérin; CIS: carcinoma in situ; HG: high grade; LG: low grade; NAC: neoadjuvant chemotherapy; NMIBC: non-muscle invasive bladder cancer; TURBT: transurethral resection of bladder tumor; UTUC: upper urinary tract urothelial carcinoma; WHO: world health organization. ^†^The risk was classified according to the Clinical Practice Guidelines for Bladder Cancer 2019 [14].

**Table 4:** Univariate and multivariate Fine-Gray competing risk regression analysis for UTUC recurrence-free survival in patients with primary NMIBC.

Variables	Univariate			Multivariate		
	HRs	95% CI	*P* value	HRs	95% CI	*P* value
Age, years						
<70	1					
≥70	0.80	0.32–2.00	0.64			
Sex						
Male	1					
Female	1.11	0.33–3.74	0.87			
Smoking history						
Never	1					
Former/Current	1.44	0.52–4.00	0.48			
Tumor size						
≤30 mm	1			1		
>30 mm	2.86	1.17–6.97	0.021	2.53	1.04–6.15	0.040
Multiplicity						
Simple	1					
Multiple	0.85	0.33–2.14	0.72			
Grade (WHO 2004)						
Low grade	1			1		
High grade	7.07	2.05–24.43	0.002	2.28	0.48–10.78	0.30
CIS						
Negative	1			1		
Positive	8.02	3.09–20.81	<0.001	4.78	1.48–15.42	0.0089
Prostate-involving CIS						
Negative	1					
Positive	1.56	0.21–11.61	0.66			
LVI						
Negative	1					
Positive	2.23	0.80–6.20	0.12			
Variant						
Negative	1					
Positive	1.68	0.24–11.99	0.60			

CIS: carcinoma in situ; HR, hazard ratio; LVI: lymphovascular invasion; NMIBC: nonmuscle invasive bladder cancer; PS: performance status; TURBT: transurethral resection of bladder tumor; UTUC: upper urinary tract urothelial carcinoma; WHO: World Health Organization.

## Data Availability

The datasets generated and/or analyzed during the current study are available from the corresponding author on reasonable request.

## Abbreviations

BCG: Bacillus Calmette-Guérin
CI: Confidence interval
CIS: Carcinoma in situ
CTU: Computed tomography urography
ECOG-PS: Eastern cooperative oncology group performance status
HG: High grade
HR: Hazard ratio
LVI: Lymphovascular invasion
NMIBC: Nonmuscle invasive bladder cancer
OS: Overall survival
SD: Standard deviation
TURBT: Transurethral resection of bladder tumor
UTUC: Upper urinary tract urothelial cancer
WHO: World Health Organization.

## Supplementary Materials

Supplementary Materials Supplementary Figure S1: cumulative incidence of UTUC recurrence-free survival after initial TURBT according to age, sex, ECOG-PS, multiplicity, prostate-involving CIS, lymphovascular invasion, variant histology, and intravesical recurrence. Survival curves of the cumulative incidence of UTUC recurrence-free survival after initial TURBT for primary NMIBC are plotted according to age (A), sex (B), ECOG-PS (C), multiplicity (D), prostate-involving CIS (E), lymphovascular invasion (F), variant histology (G), and intravesical recurrence (H).
