# Supplementary figures and images for: Routine Surveillance of Upper Urinary Tract Imaging for Diagnosing Upper Urinary Tract Urothelial Cancer Recurrence in Patients with Nonmuscle Invasive Bladder Cancer
Source: Adv Urol. 2024 May 21;2024:5894288. doi: 10.1155/2024/5894288 (PMC11132829; doi:10.1155/2024/5894288)

**Supplementary Figure S1**


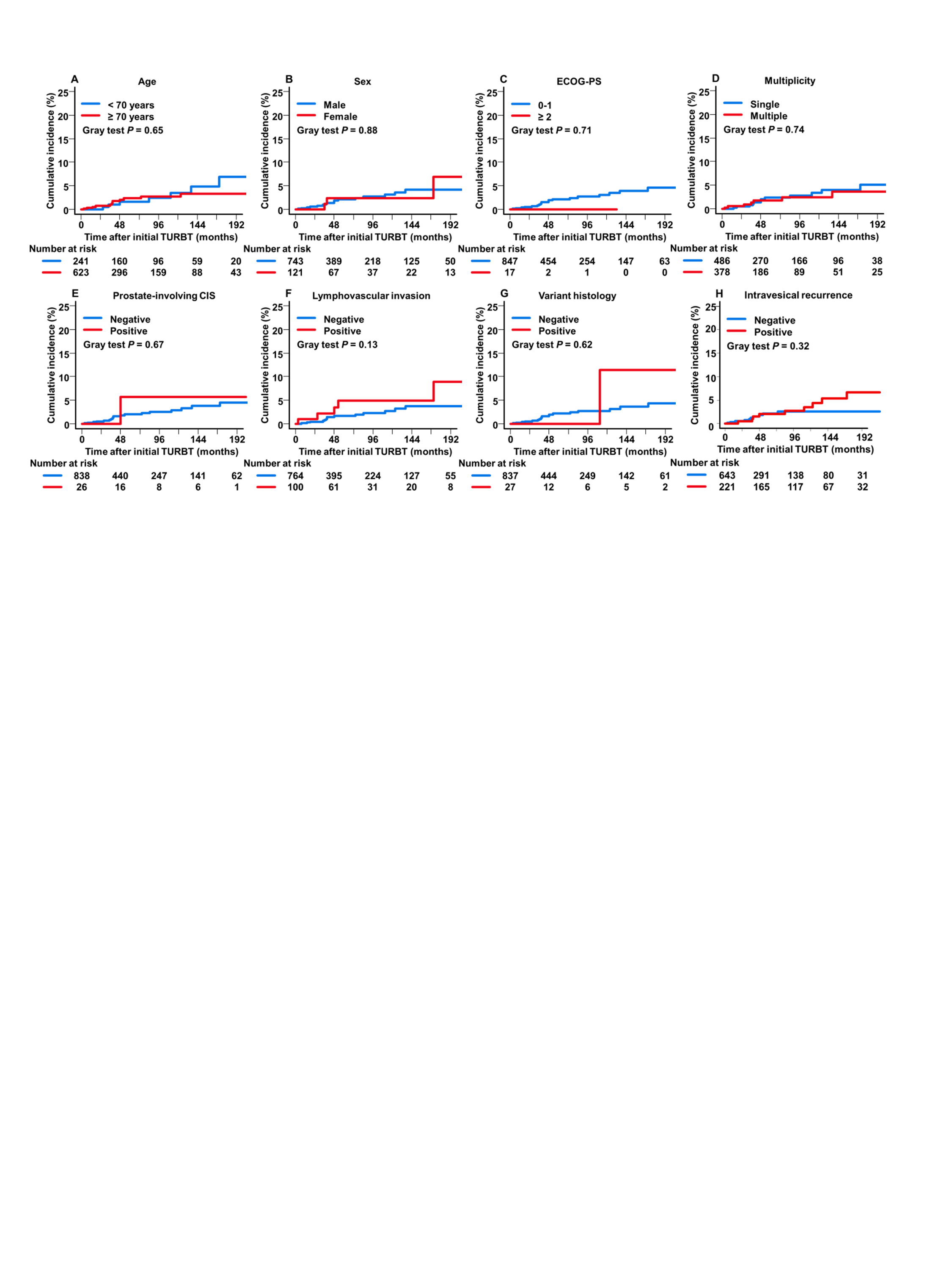

Supplement: Supplementary Materials — Supplementary Figure S1: cumulative incidence of UTUC recurrence-free survival after initial TURBT according to age, sex, ECOG-PS, multiplicity, prostate-involving CIS, lymphovascular invasion, variant histology, and intravesical recurrence. Survival curves of the cumulative incidence of UTUC recurrence-free survival after initial TURBT for primary NMIBC are plotted according to age (A), sex (B), ECOG-PS (C), multiplicity (D), prostate-involving CIS (E), lymphovascular invasion (F), variant histology (G), and intravesical recurrence (H). [file 5894288.f1.docx]
